# Clinical and Radiological Results over the Medium Term of Isolated Acetabular Revision

**DOI:** 10.1155/2014/148592

**Published:** 2014-12-28

**Authors:** Nicola Piolanti, Lorenzo Andreani, Paolo Domenico Parchi, Enrico Bonicoli, Francesco Niccolai, Michele Lisanti

**Affiliations:** 1st Orthopedic Division, Department of Translational Research and New Technologies in Medicine, University of Pisa, Via Paradisa 2, 56124 Pisa, Italy

## Abstract

Acetabular cup loosening is associated with pain, reduced function, and instability of the implant. If such event happens while the femoral implant is in a satisfactory position and is well fixed to the bone, isolated acetabular revision surgery is indicated. The aim of this single-center retrospective study was to evaluate the clinical and radiological results over the medium term (12-month follow-up mean 36, max 60) of isolated acetabular revisions surgery using a porous hemispheric revision shell matched with a cemented all-poly cup and large diameter femoral head (>32). 33 patients were enrolled. We collect any relevant data from the clinical board. Routine clinical and radiographic examinations were performed preoperatively; the postoperative follow-up was made at 1, 3, and 6 months and yearly thereafter. At the last available follow-up, we report satisfactory improvement of functional scores in all the patients; 2 patients (6.1%) showed thigh pain and only 4 hips (12.11%) presented mild groin pain; all the femoral components are well fixed and there were no potential or pending rerevisions. With bias due to the follow-up and to the retrospective design of the study, we report clinical, functional, and radiological satisfactory results.

## 1. Introduction

The most common reason for failure of total hip arthroplasty (THA) is periprosthetic osteolysis and loosening of hip implants [1]. The rate of osteolysis varies between femoral and acetabular sides, and it is more common on the acetabular side. This is why acetabular cup loosening is the main cause for revision in long-term studies [2]. This loosening usually is associated with pain, reduced function, and instability of the implant.

There are two main problems to solve when an orthopaedic surgeon has to approach acetabular revision. First of all, even with bone loss due to the loosening of the previous implant, obtain primary fixation of the new prosthesis; then reach postoperative implant stability.

This second issue could be more difficult when isolated acetabular revision is performed [3, 4].

Effectively in these cases not only does the presence of the stem limit surgical options, but also repeated surgical incision, soft tissue damage, and in some cases extended synovectomy can reduce the stability of the implant [5].

To address this risk, industries and surgeons have developed a variety of surgical hardware and strategies such as jumbo femoral heads [6], constrained acetabular liners [7] and dual-mobility cup [8].

Another way to face these problems is, in order to obtain fixation, the implantation of a shell in the better position allowed by the bone defect; then obtain stability cementing a polyethylene liner in the shell with a partially independent version and verticality.

The present study was conducted to evaluate the clinical and radiological results over the medium term (>12-month follow-up mean 36, max 60) of isolated acetabular revisions surgery using a porous hemispheric revision shell matched with a cemented all-poly cup and large diameter femoral head (>32).

## 2. Material and Methods

This single-center retrospective study was approved by our local ethical committee, and the patients gave written consent to participate. A review of our database between January 2009 and December 2012 for revision hip arthroplasty was done. In this period we performed 86 THA revisions; we selected patients that had isolated acetabular revisions with a porous hemispheric revision shell matched with a cemented all-poly cup and large diameter femoral head (>32).

Information of ages, sex, clinical history, drug treatment, and preoperative and postoperative X-ray studies were collected and recorded. The acetabular defects were classified as described by Paprosky et al. [9].

**Figure 1:**
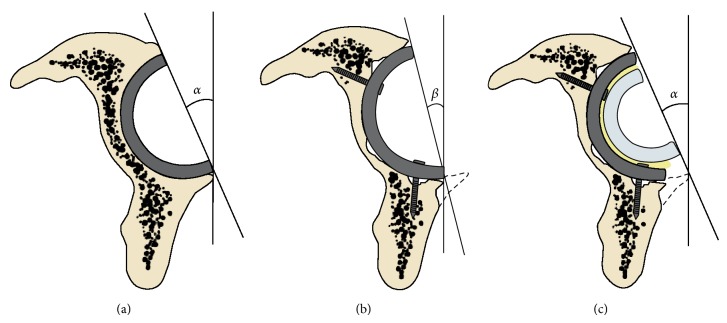
(a) *α* angle shows the correct acetabular anteversion reached after primary hip implant. (b) *β* angle obtained after revision hip surgery: the shell was implanted in the better position allowed by the bone defect. (c) During the revision surgery, in order to obtain the stability of the implant, the polyethylene liner was cemented into the shell in order to obtain the correct verticality and anteversion.

We included patients with (1) loosening or malpositions of the acetabular components, (2) a well fixed and well positioned femoral stem. We excluded (1) patients with septic loosening, (2) patients that required revision for both components, and (3) patients with monoblock stem. All the patients were operated on in the lateral decubitus position, and the surgical approach was posterolateral. We checked the stability of the stem and then we removed the acetabular cups, liners, and screws. If required before implantation of the revision components pelvic bony defects were grafted with tricalcium phosphate hydroxyapatite and morcelized bone graft. In all the index patients, the Regenerex revision shell (Biomet Warsaw, IN, USA) was implanted. This is a porous titanium construct, with multiple holes to maximize intraoperative screws fixation, designed to accept a cemented all-poly cup. During the revision surgery, the shell was implanted in the better position allowed by the bone defect; then in order to obtain the stability of the implant, the polyethylene liner was cemented into the shell in order to obtain verticality of about 45 degrees and summed of about 35 to 50 degrees of anteversion (stem plus liner) (Figure 1).

At the end of the surgical procedure, we obtained an intraoperative acceptable stability; in our opinion this is defined as 45° or more of internal rotation at 90° of flexion and 20° or more of external rotation in 10° of hyperextension. The wound was drained in each case.

In the postoperative period all the patients performed thromboembolic prophylaxis, with low molecular weight heparin; we did not use nonsteroidal anti-inflammatory drugs to prevent heterotopic periprosthetic ossification.

Partial weight bearing was allowed since the first postoperative week in the majority of patients; in some due to poor patient's bone stock we delayed it to the first radiological and clinical follow-up (six weeks) and gradually advanced it to full weight bearing. No postoperative bracing was used.

Routine clinical and radiographic examinations were performed preoperatively; the postoperative follow-up was made at 1, 3, and 6 months and yearly thereafter. Patients were scored as routinely in our practice preoperatively with the Harris Hip Score (HHS) and the Western Ontario and McMaster Universities Osteoarthritis Index (WOMAC) [10, 11]; the same scores were used during the follow-up. As routinely during clinical evaluation, we collect and report data about any relevant adverse event that occurred; in addition we also investigate if there is thigh or groin pain, any subjective perception of instability, and subsequent apprehension.

Radiographs were evaluated by two of the senior authors (Enrico Bonicoli and Paolo Domenico Parchi), with consensus attained for reporting of all measurements. The radiographic follow-up was performed in order to evaluate the position of the implant and to search for any signs of osseointegration or loosening of the components.

The de Lee and Charnley classification [12] was used; radiolucent lines were considered present if they were greater than 1 mm at their maximum width and involved any two adjacent sectors of the cup surface [13]. A horizontal or vertical change in position of at least 3 mm, or a change in abduction angle of at least 5°, was considered migration [14]. According to Brooker et al., on the last available X-ray, we also evaluated heterotopic ossification [15].

The Wilcoxon signed-rank test was used to compare preoperative and postoperative hip scores.

## 3. Results

33 patients were enrolled for this study. There were 23 women (69,7%) and 10 men (30,3%); the mean age at the revisions was 67 years (range 40–81 years); 17 were left (51,5%) and 16 (48,5%) were right. The mean clinical follow-up was 36 months (minimum 13 months, maximum 60 months); one died from unrelated illnesses; 2 patients were lost to follow-up.

The preoperative diagnoses were 26 with aseptic loosening (78,79%), 3 with implant instability (9,1%), and 4 revisions due to metallosis (12,11%).

**Figure 2:**
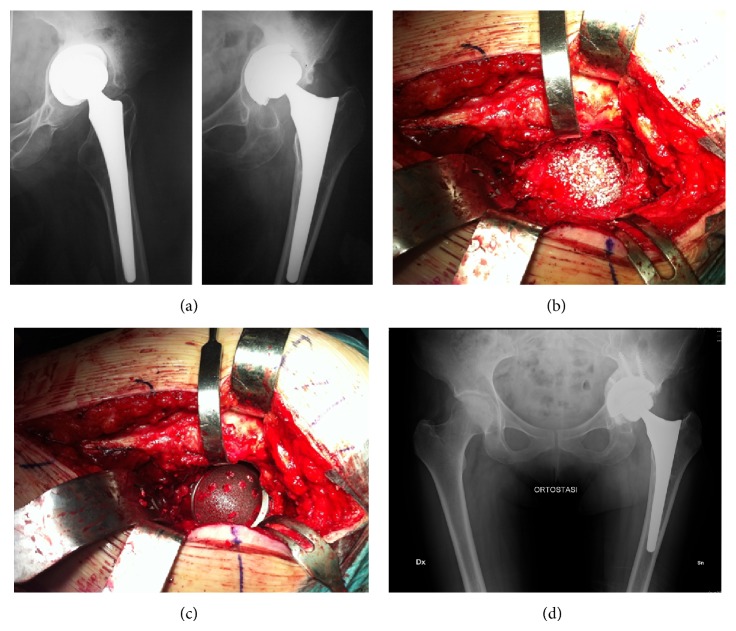
(a) Loosening of an acetabular press fit cup, stable stem. (b) Intraoperative picture showed bone defect grafted with tricalcium phosphate hydroxyapatite and morcelized bone graft. (c) Regenerex revision shell in place. (d) X-ray at the last available follow-up showing acetabular integration.

We treated 9 with type 1 defect of the Paprosky classification [9] (27%), 5 with type 2a (15%), 7 with type 2b (21%), 5 with type 2c (15%), and 7 with type 3a (21%). Cup trend was size 56, max size 68 and min size 54. In 13 patients, acetabular bone defects were grafted using tricalcium phosphate hydroxyapatite and morcelized bone graft (Figure 2); in 2 patients we used titanium augmentation. The mean number of screws used to secure the revision shells was five (from 3 to 7). The size of the femoral head was 32 (21 hips) and 36 mm (12 hips). See Table 1 for more details.

Early complications (during the in-patient stay) occurred in 4 of the 33 patients (12,11%). These included a superficial wound infection in two, deep-vein thrombosis in one, and postoperative early dislocation in one.

The postoperative early dislocation happened during a possible wrong movement of the patient from the operative room to the ward in a patient under spinal anesthesia. We reduced the dislocation with external manoeuvres and this event also at the last follow-up never happened again.

The mean WOMAC score in 25 patients improved significantly from 52.1 preoperatively to 62.27 postoperatively (*P* = 0.008). The mean Harris Hip Score improved from 59 points (range 43–74) preoperatively to 88 points (range 67–92) (*P* = 0.002). At the final follow-up, 2 patients (6,1%) showed thigh pain and only 4 hips (12.11%) presented mild groin pain; all the femoral components are well fixed and there were no potential or pending rerevisions.

**Figure 3:**
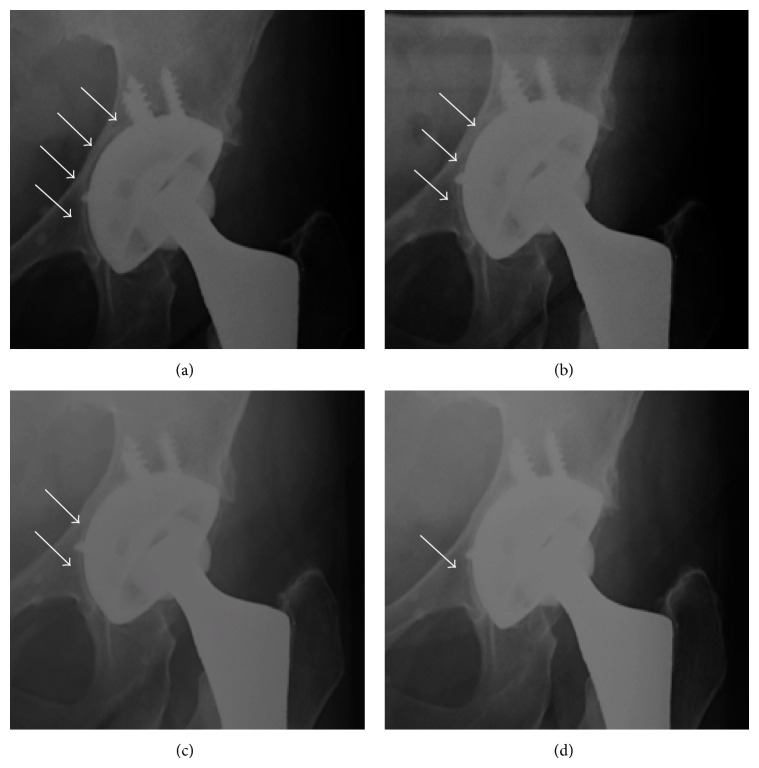
(a) Postoperative radiographs showing radiolucent lines which involved two adjacent sectors of the cup surface. ((b), (c), and (d)) Progressive osseointegration during 3, 6, and 12 months' follow-up.

With regard to the radiological evaluation, according to de Lee and Charnley [12], at the initial follow-up, we found 7 (21%) of the Regenerex implants with radiolucent lines bigger than 1 mm at their maximum width and which involved one or two adjacent sectors of the cup surface. In the last examination, five of the seven cases with radiolucent lines remaining still visible on the X-rays and seem not to be evolutive. In agreement with Schmalzried and Harris [16] and Petersen et al. [17], we attribute the filling of the gap to the formation of new bone (Figure 3).

In 3 patients osteolysis around the screws was noted, without any change of the cup orientation and without evident evolution. No change of position was found. Even in our series it is not possible to establish the influence of bone grafting for the final stabilization of the implant; when it was used, good bone osseointegration was seen.

Heterotopic ossification type 1-2 of Brooker classification [14] was found in 3 patients.

We do not report major (>2 cm) leg length discrepancy. At the most recent follow-up no one patient had experienced a further dislocation.

## 4. Discussion

In this study we evaluate the clinical and radiological results over the medium term of isolated acetabular revision with a porous hemispheric revision shell matched with a cemented all-poly cup and large diameter femoral head (>32).

Besides the lack of a control group there are some limitations in this study, such as the short follow-up of same patients and the retrospective design.

However we think that our study can improve the literature's knowledge about this issue also because as reported in the Australian registry, the revision of the acetabular component is the most common cause of repeated surgery in total hip replacement [18]. Isolated acetabular revision is indicated when an acetabular implant is associated with pain, reduced function, instability, or loosening, while the femoral implant is in satisfactory position and is well fixed to bone. The benefits of leaving the femoral component in place include reduced operating time, less blood loss, and preservation of bone stock [19]. Doubtless the presence of the femoral implant limits the surgical exposure, making access to the acetabulum and treatment of bone defects challenging and increasing the risk of postoperative dislocation.

The author's choice to face the problem of achieving fixation of the implant is to choose porous materials such as Regenerex during revision surgery. The advantage of these materials is not only to provide a primary stability due to scratch fit but also to allow long-term implant stability related to bone ingrowth (osteoconductivity) [20], which is important especially in revision surgery when bone quality is poor [21, 22]. Another advantage of such technique is to implant the shell in the better position allowed by the bone defect reaching the better possible primary stability and then cementing the polyethylene liner with version and verticality partially independent (Figure 2).

In this way it is possible to obtain the correct geometry of the revised implant (verticality of about 45 degrees and summed of about 35° to 50 degrees of anteversion, stem plus liner) issue that is important to reduce the risk of dislocation which has been associated with hip revision surgery and rises in isolated acetabular revision.

The author's opinion supported by biomechanical tests and several clinical series with short-term follow-up of liner cementation is that such technique is sure. The cemented polyethylene liner was found to have an initial fixation strength exceeding that of the conventional locking mechanism if 2 and 4 mm thick cement mantles were built up around the liner [18]. It is undisputed that large femoral heads improve stability of the hip implant by increasing the excursion before dislocation can occur [23]. The literature does not clarify whether any preoperative variables influence pain relief or functional scores [24]; however we report satisfactory improvement of functional scores (HHS improves from 59 to 88 and WOMAC from 52,1 to 62,27) with only 4 patients (12,11%) complaining of mild groin pain and 2 (6,1%) patients with thigh pain. We do not report major (>2 cm) leg length discrepancy. The only one dislocation (3%) is in-line or even better compared with the results reported from other authors [5–7, 24]; such dislocation appears to be correlated to a wrong patient transfer from the operative to the to the ward matched with spinal anesthesia.

Schneider et al. [5] had 10 cases of dislocation (10.4%) in a series of 96 revisions with a reconstruction cage and a cemented dual-mobility cup. Della Valle et al. [7] in their series of 55 cases at a mean follow-up of 3,6 years reported a dislocation rate of 16%. Lawless et al. [24] reported a dislocation rate of 0% at 6,4 years after revision but with a 7,3% of reoperation due to aseptic loosening. With bias due to the follow-up and to the retrospective design of the study, in our series complications were low and we did not report reoperations, not only for the acetabular component but also for the stem; such results compare favorably with previous report [8, 25]. In our opinion, selective acetabular revision with a porous hemispheric revision shell matched with a cemented all-poly cup and large diameter femoral head is a reliable alternative with excellent clinical success over the medium term. Precise check of the preoperative X-rays and a careful evaluation of the intraoperative stem stability are mandatory for such results. Nevertheless more detailed and scrupulous evaluation with long-term prospective studies is needed.

## Figures and Tables

**Table 1:** Relevant data of the revised implant and data about the new components.

Patient	Side	Sex	Age	Previous implant	Implant	Explanted head	Diagnosis	Cup	Paprosky	Size cup	Screw number	Head	Graft
1	Right	F	72	Press-fit	Uncemented	28 met	Aseptic loosening	Regenerex	2 B	54	4	Cer 32	Yes
2	Right	F	54	Press-fit	Uncemented	36 cer	Aseptic loosening	Regenerex	2 C	56	5	Cer 36	Yes
3	Left	F	58	Press-fit	Uncemented	28 met	Implant instability	Regenerex	2 A	54	4	Cer 32	Yes
4	Right	F	64	Cemented	Cemented	28 met	Aseptic loosening	Regenerex	2 A	54	4	Cer 32	No
5	Left	F	61	Press-fit	Uncemented	36 met	Metallosis	Regenerex	1	54	4	Cer 32	No
6	Right	F	73	Press-fit	Uncemented	36 cer	Aseptic loosening	Regenerex	2 A	54	5	Cer 32	No
7	Right	F	77	Treated	Uncemented	36 cer	Aseptic loosening	Regenerex	1	58	5	Cer 32	No
8	Left	M	75	Cemented	Uncemented	28 met	Aseptic loosening	Regenerex	2 C	62	5	Cer 36	Yes
9	Left	M	73	Cemented	Cemented	28 met	Aseptic loosening	Regenerex	3 A	56	5	Cer 32	Yes
10	Left	F	74	Press-fit	Uncemented	28 cer	Aseptic loosening	Regenerex	2 B	60	5	Cer 32	Yes
11	Left	M	64	Press-fit	Uncemented	36 cer	Implant instability	Regenerex	1	58	4	Cer 32	No
12	Left	F	46	Press-fit	Uncemented	28 cer	Aseptic loosening	Regenerex	2 B	54	4	Cer 32	Yes
13	Left	M	78	Press-fit	Uncemented	28 cer	Aseptic loosening	Regenerex	2 B	62	5	Cer 36	No
14	Right	M	64	Treated	Uncemented	36 cer	Aseptic loosening	Regenerex	2 A	58	5	Cer 36	No
15	Right	F	71	Cemented	Uncemented	36 cer	Aseptic loosening	Regenerex	3 A	56	4	Cer 32	Yes
16	Right	M	64	Press-fit	Uncemented	32 cer	Aseptic loosening	Regenerex	2 B	56	5	Cer 32	No
17	Right	F	76	Cemented	Uncemented	36 cer	Aseptic loosening	Regenerex	2 B	56	6	Cer 32	No
18	Left	F	40	Press-fit	Uncemented	36 cera	Aseptic loosening	Regenerex	1	50	3	Cer 32	No
19	Left	M	74	Treated	Uncemented	36 cer	Aseptic loosening	Regenerex	2 B	62	6	Cer 36	No
20	Left	M	67	Press-fit	Uncemented	28 met	Implant instability	Regenerex	1	54	6	Cer 32	No
21	Right	M	58	Press-fit	Uncemented	50 met	Metallosis	Regenerex	1	58	5	Cer 36	No
22	Right	F	64	Press-fit	Uncemented	50 met	Metallosis	Regenerex	1	56	5	Cer 36	No
23	Left	F	72	Treated	Uncemented	32 cer	Aseptic loosening	Regenerex	2 C	60	8	Cer 36	Yes
24	Right	F	79	Press-fit	Cemented	22 met	Aseptic loosening	Regenerex	3 A	58	6	Cer 32	Yes
25	Right	F	58	Press-fit	Uncemented	32 cer	Aseptic loosening	Regenerex	3 A	56	7	Cer 32	Yes
26	Left	F	74	Press-fit	Uncemented	36 met	Aseptic loosening	Regenerex	2 A	54	5	cer 32	No
27	Right	M	74	Press-fit	Uncemented	22 met	Aseptic loosening	Regenerex	1	68	6	Cer 36	No
28	Left	F	72	Press-fit	Uncemented	36 cer	Aseptic loosening	Regenerex	3 A	56	7	Cer 32	Titanium augmentation
29	Left	F	81	Press-fit	Cemented	28 met	Aseptic loosening	Regenerex	3 A	56	5	Cer 32	Titanium augmentation
30	Right	F	63	Treated	Uncemented	36 cer	Aseptic loosening	Regenerex	2 C	60	5	Cer 36	No
31	Left	F	75	Cemented	Uncemented	28 met	Aseptic loosening	Regenerex	2 C	60	7	Cer 36	Yes
32	Right	F	75	Press-fit	Uncemented	28 met	Aseptic loosening	Regenerex	3 A	54	6	Cer 32	Yes
33	Left	F	59	Press-fit	Uncemented	40 met	Metallosis	Regenerex	1	58	6	Cer 36	No
